# Fatty liver index as a predictor for type 2 diabetes in subjects with normoglycemia in a nationwide cohort study

**DOI:** 10.1038/s41598-021-95546-x

**Published:** 2021-08-12

**Authors:** E. García-Escobar, S. Valdés, F. Soriguer, J. Vendrell, I. M. Urrutia-Etxebarria, C. Maldonado-Araque, E. Ortega, P. Ocón, E. Montanya, E. Menéndez, A. Lago-Sampedro, T. González-Frutos, R. Gomis, A. Goday, S. García-Serrano, J. L. Galán-García, C. Castell, E. Bordiú, R. Badía, G. Aguilera-Venegas, J. Girbés, S. Gaztambide, E. Delgado, F. J. Chaves, L. Castaño, A. Calle-Pascual, G. Rojo-Martínez, J. Franch-Nadal

**Affiliations:** 1 Spanish Biomedical Research Network in Diabetes and Associated Metabolic Disorders (CIBERDEM), Madrid, Spain; 2 Endocrinology and Nutrition Department, Biomedical Research Institute of Malaga (IBIMA), Regional University Hospital of Malaga, Málaga, Spain; 3 Rovira i Virgili University; Department of Endocrinology and Nutrition, Hospital Universitario Joan XXIII, Institut d’Investigacions Sanitaries Pere Virgili, Tarragona, Spain; 4 Cruces University Hospital, BioCruces Bizkaia, UPV/EHU, Endo-ERN, Barakaldo, Spain; 5 Spanish Biomedical Research Network in Rare Diseases (CIBERER), Madrid, Spain; 6 Department of Endocrinology and Nutrition, August Pi i Sunyer Biomedical Research Institute – IDIBAPS, Hospital Clínic of Barcelona, Barcelona, Spain; 7 Spanish Biomedical Research Network in Physiopathology of Obesity and Nutrition (CIBEROBN), Barcelona, Spain; 8 General Laboratory, Regional University Hospital of Malaga, Málaga, Spain; 9 Bellvitge Biomedical Research Institute (IDIBELL), University of Barcelona, Bellvitge University Hospital, Barcelona, Spain; 10 Department of Endocrinology and Nutrition, Central University Hospital of Asturias/University of Oviedo, Health Research Institute of the Principality of Asturias (ISPA), Oviedo, Spain; 11 Endocrinology and Nutrition Department, Hospital del Mar/Medicine Departament, Univeristat Autonoma de Barcelona, Barcelona, Spain; 12 Department of Applied Mathematics, Malaga University, Málaga, Spain; 13 Department of Health, Public Health Agency of Catalonia, Barcelona, Spain; 14 Department of Endocrinology and Nutrition, San Carlos University Hospital of Madrid, Madrid, Spain; 15 Diabetes Unit, Hospital Arnau de Vilanova, Valencia, Spain; 16 Genomic and Genetic Diagnosis Unit, Research Foundation of Valencia University Clinical Hospital-INCLIVA, Valencia, Spain; 17 EAP Raval Sud, Catalan Institute of Health, GEDAPS Network, Primary Care, Research Support Unit (IDIAP – Jordi Gol Foundation), Barcelona, Spain

**Keywords:** Predictive markers, Type 2 diabetes, Risk factors

## Abstract

Our aim was to evaluate whether fatty liver index (FLI) is associated with the risk of type 2 diabetes (T2DM) development within the Spanish adult population and according to their prediabetes status; additionally, to examine its incremental predictive value regarding traditional risk factors. A total of 2260 subjects (Prediabetes: 641 subjects, normoglycemia: 1619 subjects) from the Di@bet.es cohort study were studied. Socio-demographic, anthropometric, clinical data and survey on habits were recorded. An oral glucose tolerance test was performed and fasting determinations of glucose, lipids and insulin were made. FLI was calculated and classified into three categories: Low (< 30), intermediate (30–60) and high (> 60). In total, 143 people developed diabetes at follow-up. The presence of a high FLI category was in all cases a significant independent risk factor for the development of diabetes. The inclusion of FLI categories in prediction models based on different conventional T2DM risk factors significantly increase the prediction power of the models when all the population was considered. According to our results, FLI might be considered an early indicator of T2DM development even under normoglycemic condition. The data also suggest that FLI could provide additional information for the prediction of T2DM in models based on conventional risk factors.

## Introduction

Non-alcoholic fatty liver disease (NAFLD) is characterized by increased fat storage in form of triglycerides in the liver (exceeding 5% of its weight) in absence of excessive alcohol consumption^1^. It is the most frequent liver disease in Western countries with an estimated overall worldwide prevalence in the adult population of about 25%^2^, value that is substantially increased in subjects with type 2 diabetes (T2DM)^2^. A recent study in the United States population has reported the presence of NAFLD in up to 78–85% of patients with T2DM, based on the use of different non-invasive markers of liver steatosis^3^. Specifically in Spain, the prevalence of NAFLD has been estimated to be 26% among the adult population^4^.

NAFLD is associated with several liver disorders, terminal liver failure and hepatocellular carcinoma^5^, cardiovascular disease^6^ and it is considered as the hepatic manifestation of metabolic syndrome^7,8^. Additionally, increasing epidemiological evidence suggests a bidirectional relationship between NAFLD and T2DM may be linked by insulin resistance^9–11^. This concept would be in line with several studies that have consistently shown that NAFLD is an independent risk factor for prediabetes or T2DM^12–14^. Given the progressive nature of the disease and its risk of adverse consequences, health care providers are strongly advised to screen for NAFLD in all patients with diabetes and to be more proactive in their management^11^.

The gold standard for diagnosis of NAFLD is the liver biopsy, which is an invasive technique only justified in severe liver disease^15^. 1H-magnetic resonance spectroscopy (MRS) allows a quantification of hepatocellular lipid content and an exact diagnosis of steatosis^15^, while ultrasound and computed tomography provide semi-quantitative estimations^16^. These techniques are time-consuming, expensive and often unavailable in the daily routine. As an alternative, several panels or ‘scores’ consisting of combinations of anthropometric and biochemical parameters have been developed for the diagnosis and quantification of steatosis. Among all of them, the fatty liver index (FLI) has gained popularity^17,18^. FLI is a non-invasive and well-established method for the diagnosis of fatty liver validated against ultrasound^17–20^ and MRS^21^ in both Asian and Western populations. It includes 4 variables: body mass index (BMI), waist circumference, serum triglycerides and serum gamma-glutamyl transferase (GGT)^18^.

Several previous studies, have reported that FLI is a predictor for the development of T2DM in the general population^22–24^ and in subjects who have an especially higher risk of DM2 (prediabetic status)^14,25–27^. Accordingly, FLI could be potentially useful to further identify those who are at higher risk of conversion to new onset of T2DM in order to initiate primary prevention efforts with aggressive lifestyle management^28^. Nevertheless, the additive predictive value of FLI beyond the conventional risk factors of T2DM has been investigated less and with inconclusive results^23,25^. In the same way, it is unclear whether FLI is associated with the development of T2DM in individuals without prediabetes who might be at lower risk for incident T2DM. To our knowledge, only one study performed in a Japanese population has reported the potential utility of FLI as a predictor for T2DM development in subjects without hyperglycemia^29^.

Therefore, the aim of this study was to evaluate whether fatty liver estimated by FLI is associated with the risk of T2DM within the Spanish adult population with and without prediabetes. In addition, we also examined the incremental predictive value of FLI in diagnosing individuals who will develop new-onset T2DM over 7.5 years of follow-up in the Spanish general adult population and according to the prediabetes status.

## Results

### Baseline characteristics of the population

The baseline characteristics of the subjects according to FLI categories in the overall population and the groups of subjects with and without prediabetes are presented in Table 1. For all the cases, groups of subjects in the low FLI category were younger and with a lower percentage of men than the rest of FLI categories. In general, anthropometric variables, clinical parameters and lifestyle factors were different according to FLI categories for all the studied population groups.

**Table 1:** Baseline characteristics of the subjects according to FLI categories.

Variable	Overall population (2260)			*p*	Non-prediabetes (1619)			*p*	Prediabetes (641)			*p*
	Low FLI (958)	Intermediate FLI (571)	High FLI (731)		Low FLI (822)	Intermediate FLI (407)	High FLI (390)		Low FLI (136)	Intermediate FLI (164)	High FLI (341)	
Age (years)	42.61 ± 13.77	50.56 ± 14.45	52.87 ± 13.92	< 0.001	41.65 ± 13.52	49.19 ± 14.14	51.20 ± 14.26	< 0.001	48.30 ± 13.91	53.98 ± 14.70	54.71 ± 13.35	< 0.001
Sex (% men)	21.8	48.3	56	< 0.001	21.5	49.1	56.2	< 0.001	23.5	46.3	44.3	< 0.001
BMI (kg/m^2^)	23.92 ± 2.63	27.79 ± 2.43	32.14 ± 4.20	< 0.001	23.78 ± 2.61	27.66 ± 2.40	31.86 ± 4.08	< 0.001	24.78 ± 2.63	28.12 ± 2.47	32.44 ± 4.31	< 0.001
Waist (cm)	81.03 ± 8.20	94.11 ± 6.13	105.06 ± 9.38	< 0.001	80.53 ± 8.22	94.11 ± 6.15	104.14 ± 9.31	< 0.001	84.05 ± 7.40	94.12 ± 6.11	106.11 ± 9.35	< 0.001
Cholesterol (mg/dl)	186.68 ± 37.64	204.35 ± 37.80	207.86 ± 37.07	< 0.001	184.98 ± 37.07	204.43 ± 37.85	207.54 ± 35.91	< 0.001	196.81 ± 39.49	204.15 ± 37.80	208.45 ± 38.60	< 0.001
cHDL (mg/dl)	57.44 ± 13.52	52.57 ± 11.55	47.77 ± 10.95	< 0.001	57.07 ± 13.55	51.97 ± 11.29	47.32 ± 10.92	< 0.001	59.63 ± 13.14	54.04 ± 12.07	48.20 ± 11.07	< 0.001
cLDL (mg/dl)	96.49 ± 27.52	112.30 ± 29.03	115.17 ± 27.76	< 0.001	95.36 ± 26.97	112.94 ± 29.00	114.75 ± 26.75	< 0.001	103.13 ± 29.89	110.70 ± 29.14	115.65 ± 28.90	< 0.001
Triglycerides (mg/dl)	79.34 ± 28.18	110.50 ± 42.72	159.36 ± 97.81	< 0.001	78.81 ± 27.90	110.93 ± 43.11	105.84 ± 60.05	< 0.001	82.86 ± 29.75	109.44 ± 41.87	131.38 ± 91.03	< 0.001
Glucose (mg/dl)	86.98 ± 11.47	93.50 ± 10.11	97.31 ± 10.86	< 0.001	84.55 ± 9.98	89.19 ± 7.45	90.45 ± 7.22	< 0.001	101.65 ± 8.57	104.20 ± 7.61	105.13 ± 8.84	0.38
Insulin (mUI/ml)	6.28 ± 4.09	8.24 ± 3.72	11.65 ± 6.05	< 0.001	6.10 ± 3.82	7.83 ± 3.39	10.39 ± 4.66	< 0.001	7.36 ± 5.37	9.26 ± 4.29	13.12 ± 7.07	< 0.001
HOMA-IR	1.37 ± 1.01	1.92 ± 0.94	2.84 ± 1.66	< 0.001	1.29 ± 0.88	1.73 ± 0.78	2.33 ± 1.09	< 0.001	1.86 ± 1.49	2.39 ± 1.13	3.43 ± 1.98	< 0.001
AST (U/l)	15.88 ± 5.13	17.79 ± 5.26	20.09 ± 8.97	< 0.001	15.80 ± 5.26	17.75 ± 5.34	19.79 ± 8.52	< 0.001	16.52 ± 4.58	17.87 ± 5.06	20.42 ± 9.46	0.002
ALT (U/l)	11.36 ± 5.81	13.93 ± 7.15	18.50 ± 13.48	< 0.001	11.26 ± 5.93	14.13 ± 7.62	18.01 ± 11.73	< 0.001	11.98 ± 4.92	13.43 ± 5.81	19.06 ± 15.26	< 0.001
Hypertension (%)	26	54.4	68.6	< 0.001	23.8	47.9	63.2	< 0.001	39.0	60.1	74.8	< 0.001
Family history of T2DM (%)	53	49.5	52	0.43	52.4	49.1	48.6	0.36	56.4	50.6	55.9	0.49
Adherence to Mediterranean diet (%)	33.2	37.8	36.9	0.12	32.2	38.9	35.3	0.07	39.1	35.2	38.7	0.71
Steatogenic drugs (%)	7.6	9.5	11.1	0.05	6.9	9.1	9.0	0.26	11.7	10.3	13.5	0.59
Smoking (%)	26.4	27.5	23.4	< 0.001	28.1	29.7	24.1	< 0.001	16.2	22.0	22.6	< 0.001
**Educational level (%)**												
< Primary	3.3	10.2	12.6	< 0.001	3.2	9.3	9	< 0.001	4.4	12.2	16.7	0.001
Primary + secondary	72.4	73.6	76.3		72	73.2	79.5		75.0	74.4	72.7	
University	24.2	16.3	11.1		24.8	17.4	11.5		20.6	13.4	10.6	
**Alcohol consumption**												
Never	24.6	21.6	22.8	0.001	25.7	20.9	22.3	0.002	17.6	23.3	23.5	0.55
Low	12.7	8.9	8.2		13.3	9.1	8.2		9.6	8.6	8.2	
Moderate	49.3	53.3	49.7		49.1	53.3	51.0		50.7	53.4	48.1	
High	13.4	16.1	19.3		11.9	16.7	18.5		22.1	14.7	20.2	
**IPAQ**												
Low	37.3	37.4	25.3	< 0.001	37.8	40.5	47.9	< 0.001	34.6	42.3	51.9	0.001
Moderate	41.1	31.1	27.9		37.4	31.0	31.4		37.5	31.3	33.1	
High	49.8	32.2	17.9		24.8	28.5	20.6		27.9	26.4	15.0	

*p* = signification level of sex, age and BMI adjusted ANOVA or McNemar test.

Compared to the low FLI category, individuals in the high FLI category had higher fasting glucose levels (FGL) except in the group with prediabetes, where no differences in FPG according to FLI categories were found. No differences in the family history of T2DM or in the adherence to the Mediterranean diet were found according to FLI categories for any group.

### New onset of T2DM

143 people developed T2DM after 7.5 years of follow-up, among them 106 had prediabetes at baseline. The proportions of subjects who develop T2DM were significantly different according to the FLI categories in all the studied groups (Overall population: 1.77% within low FLI category, 4.90% within intermediate FLI category and 13.04% within high FLI category. Subjects with normoglycemia: 0.73% within low FLI category, 2.7% within intermediate FLI category and 5.13% within high FLI category. Subjects with prediabetes: 8.08% within low FLI category, 10.36% within intermediate FLI category and 22.07% within high FLI category. *p* < 0.001 for all cases measured by a Chi-Square test.). Proportion of subjects who develop T2DM within each FLI category according to sex is showed in Supplementary Table S1.

### FLI as a T2DM development biomarker

We analyzed incidence rate ratios (RR) of FLI and each component (BMI, waist circumference, serum triglycerides and GGT) for new-onset of T2DM in overall population. As a result, we found that FLI and its components were significantly associated to incident T2DM (RR[95% CI]: FLI = 1.03[1.02–1.3]; BMI = 1.12[1.09–1.15]; waist circumference = 1.05[1.04–1.06]; triglycerides = 6.90[3.60–13.20] and GGT = 4.08[2.86–5.84]. *p* < 0.001 in all the cases).

Multivariate analyses for the development of T2DM at 7.5 years of follow-up in overall population (Table 2) and in subjects classified according to their prediabetes status at baseline (Table 3) were also performed. Results from these multivariate models adjusted by different confounding variables showed that the presence of a high FLI category was in all cases a significant independent risk factor for the development of T2DM with a significant p for trend for the increased incidence RR associated with higher FLI categories in overall population (Table 2), and also in subjects under normoglycemia or prediabetes conditions (Table 3). Significant independent associations remained when FLI was considered as continuous variable (Supplementary Table S2).

**Table 2:** Incidence rate ratios and 95% confidence intervals of Poisson robust multivariate regression models for the risk of incident T2DM after 7.5 years of follow-up according to FLI categories in the general population.

	Low FLI	Intermediate FLI	High FLI	*p*^a^	*p*^b^
Base model: age, sex, fasting glucose and family history of T2DM	RC	1.54 (0.83–2.85)	3.16 (1.81–5.52)	< 0.001	< 0.001
Base model + HOMA-IR	RC	1.49 (0.80–2.78)	2.93 (1.63–5.27)	< 0.001	< 0.001
Base model + total and HDL and LDL cholesterol + dyslipidemia treatment	RC	1.41 (0.76–2.62)	2.69 (1.53–4.71)	0.001	< 0.001
Base model + AST + ALT + esteatogenic medication	RC	1.50 (0.80–2.78)	2.99 (1.70–5.26)	< 0.001	< 0.001
Base model + hypertension	RC	1.48 (0.79–2.78)	3.15 (1.76–5.63)	< 0.001	< 0.001
Base model + alcohol consumption + educational level + smoking habits + Mediterranean diet adherence + physical activity	RC	1.47 (0.80–2.73)	3.13 (1.79–5.47)	< 0.001	< 0.001

*p*^a^ = Significance level in the regression model.

*p*^b^ = Significance level in the *p* for trend test.

*RC* reference category.

**Table 3:** Incidence rate ratios and 95% confidence intervals of Poisson robust multivariate regression models for the risk of incident T2DM after 7.5 years of follow-up according to FLI categories and prediabetes status.

	Low FLI	Intermediate FLI	High FLI	*p*^a^	*p*^b^
**Subjects with normoglycemia (1619)**					
Base model: age, sex, fasting glucose and family history of T2DM	RC	2.40 (0.80–7.21)	4.10 (1.48–11.33)	< 0.01	< 0.01
Base model + HOMA-IR	RC	2.34 (0.79–6.95)	3.75 (1.30–10.81)	0.01	0.01
Base model + total and HDL and LDL cholesterol + dyslipidemia treatment	RC	2.43 (0.79–7.44)	3.90 (1.48–10.26)	< 0.01	< 0.01
Base model + AST + ALT + steatogenic medications	RC	2.35 (0.78–7.12)	3.69 (1.31–10.39)	0.01	< 0.01
Base model + hypertension	RC	2.24 (0.71–7.03)	4.13 (1.45–11.76)	< 0.01	< 0.01
Base model + alcohol consumption + educational level + smoking habits + Mediterranean diet adherence + physical activity	RC	2.21 (0.73–6.73)	4.31 (1.54–12.09)	< 0.01	< 0.01
**Subjects with prediabetes (641)**					
Base model: age, sex, fasting glucose and family history of T2DM	RC	1.05 (0.51–2.17)	2.28 (1.24–4.18)	< 0.01	< 0.01
Base model + HOMA-IR	RC	1.03 (0.49–2.15)	2.17 (1.14–4.15)	0.02	< 0.01
Base model + total and HDL and LDL cholesterol + dyslipidemia treatment	RC	0.95 (0.46–1.93)	1.88 (1.01–3.50)	0.05	0.01
Base model + AST + ALT + steatogenic medications	RC	1.04 (0.50–2.13)	2.28 (1.24–4.20)	< 0.01	< 0.01
Base model + hypertension	RC	1.06 (0.51–2.20)	2.31 (1.22–4.39)	0.01	< 0.01
Base model + alcohol consumption + educational level + smoking habits + Mediterranean diet adherence + physical activity	RC	1.05 (0.52–2.15)	2.23 (1.21–4.08)	0.01	< 0.01

*p*^a^ = Significance level in the regression model.

*p*^b^ = Significance level in the *p* for trend test.

*RC* reference category.

Separate analyses by sex returned similar results in overall population (Supplementary Table S3). When population is split by prediabetes status, the RR values for the association between FLI and T2DM incidence were similar between men and women in all regression models, both in subjects with normoglicemia and prediabetes (Supplementary Table S4); nevertheless, associations remained significant mainly in the group of women with normoglicemia where the sample size was also higher.

### Predictive value of FLI

To compare the predictive value of FLI for the risk of new cases of T2DM regarding the individual variables include in its calculation, we performed comparisons of receiver operating (ROC) curves of these prediction models. Analysis of these ROC curves showed that the AUC was significantly increased in the model with FLI versus the models with its components (Supplementary Figure S1).

Additionally, we performed ROC curves for new cases of T2DM of two proposed models which include or not the FLI categories. The first proposed model (PM1) was based on the conventional risk factors for T2DM development age, sex, FGL, background of T2DM, and insulin resistance risk category (HOMA-IR). The second proposed model (PM2) was based on PM1 but excluding HOMA-IR considering the difficulties to determine HOMA values routinely in clinical practice.

**Figure 1:**
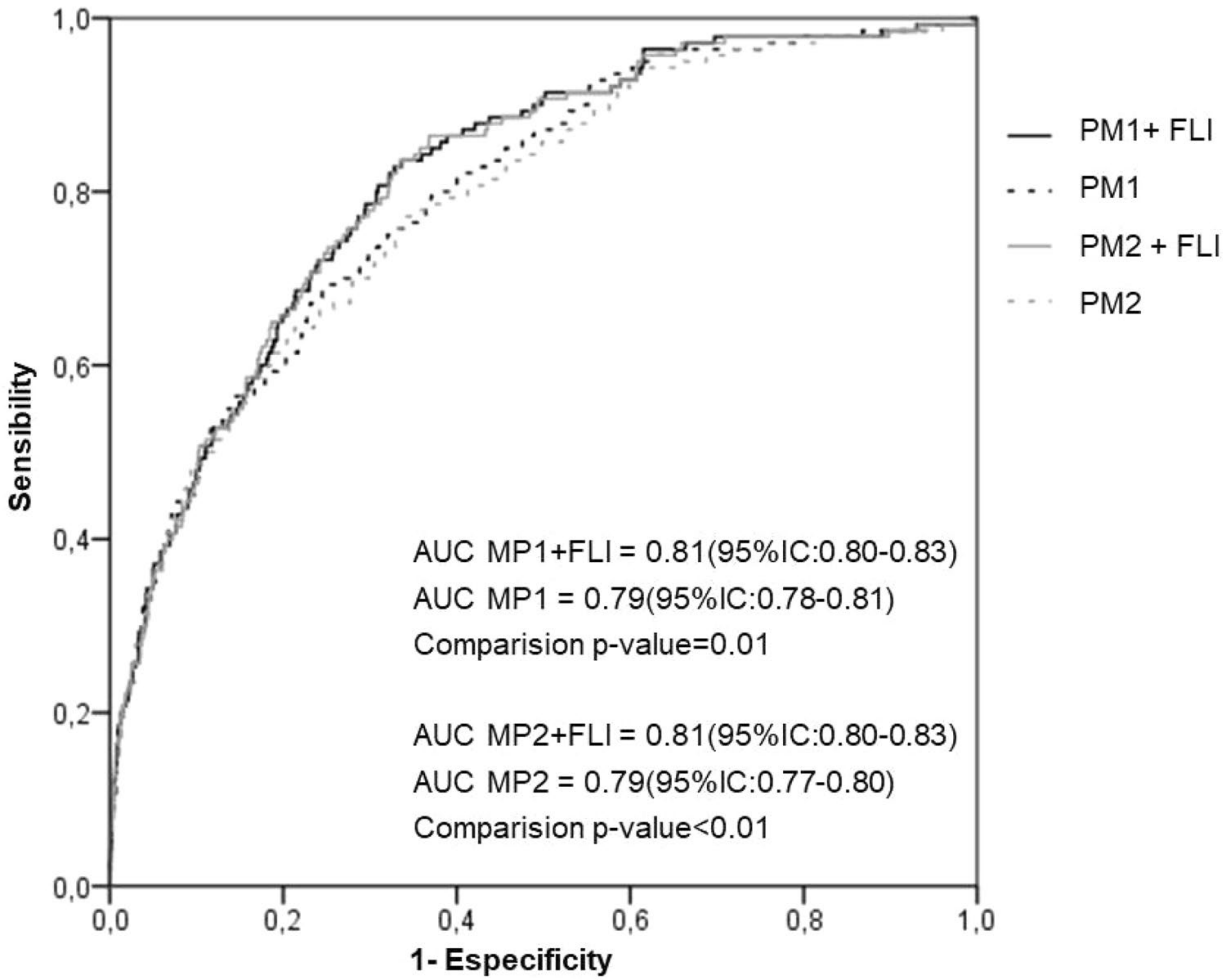
Comparison of area under the receiver-operating characteristic curve (AUC) for incident T2DM in overall population according to adding or not FLI to the conventional T2DM prediction risk models PM1 (age, sex, family history of T2DM, fasting glucose level and insulin resistance risk category) and PM2 (age, sex, family history of T2DM and fasting glucose level).

The addition of FLI categories to both PM1 and PM2 models in the overall population is shown in Fig. 1. The AUC for predicting future incidence of T2DM significantly increased when FLI was added to both diabetes prediction models (Fig. 1). We also assessed whether the addition of FLI to the conventional T2DM prediction models can improve the predictive ability for new-onset T2DM using NRI and IDI^30^. We found that the IDI values were − 0.010 (95% CI: − 0.016 to − 0.004, *p* = 0.01) for the PM1 model, and − 0.016 (95% CI: − 0.027 to − 0.026, *p* = 0.02) for the PM2 model. Additionally, the category-free NRI were 0.066 (standard error = 0.021, z-score = 2.004, *p* = 0.045) and 0.07 (standard error = 0.027, z-score = 2.57, *p* = 0.001) for the PM1 and PM2 models respectively. Thus, the addition of FLI to these basic diabetes risk models correctly reclassified 6.6% and 7% more cases respectively in the overall study population.

**Figure 2:**
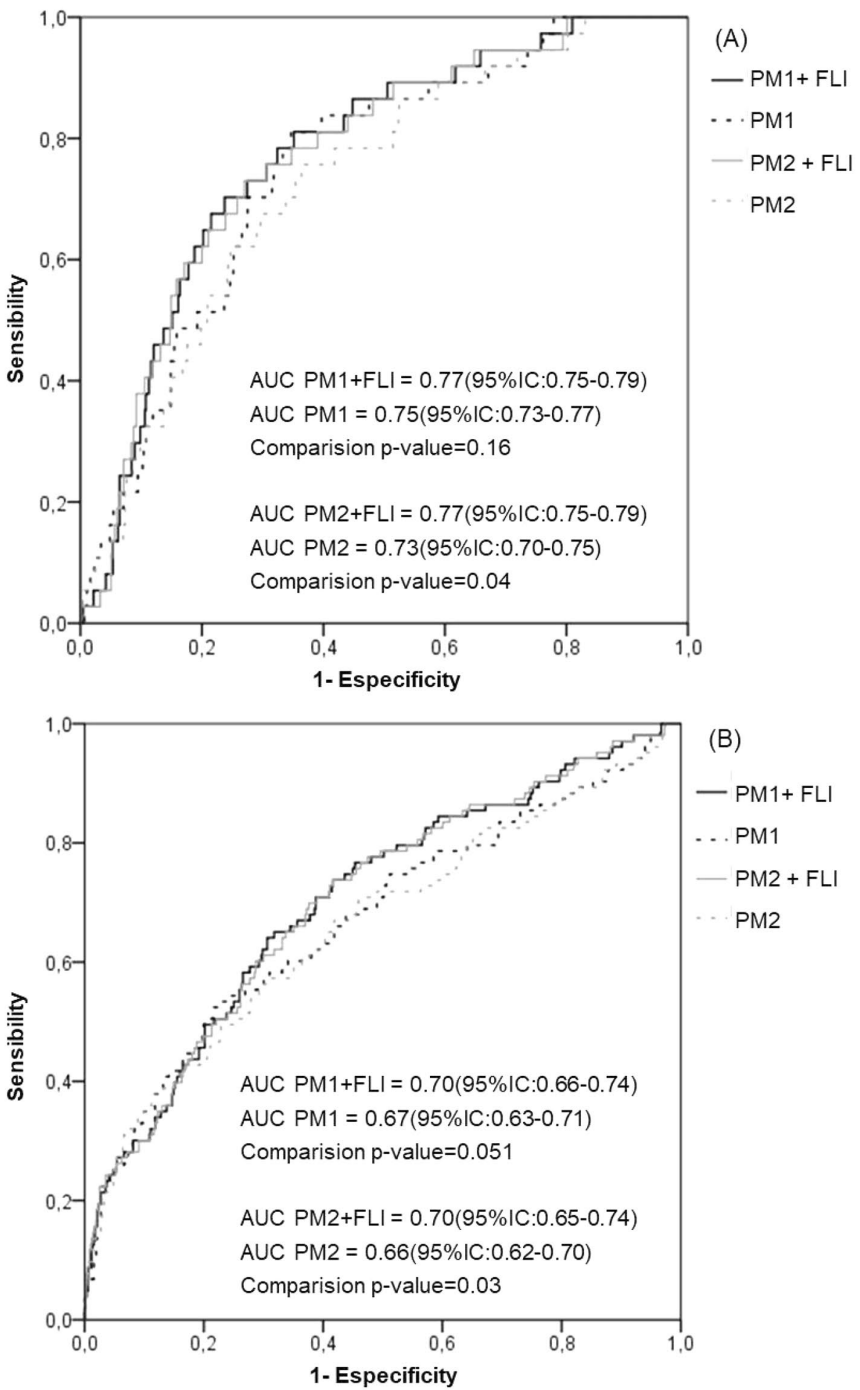
Comparison of area under the receiver-operating characteristic curve (AUC) for incident T2DM in subjects with and without prediabetes according to adding or not FLI to the conventional T2DM prediction risk models PM1 (age, sex, family history of T2DM, fasting glucose level and insulin resistance risk category) and PM2 (age, sex, family history of T2DM and fasting glucose level).

Alternatively, when the prediabetes status was considered, the inclusion of the FLI categories in the PM1 model slightly increased the AUC with no significant differences independently of the prediabetes status (Fig. 2). However, the inclusion of FLI categories in the model excluding insulin resistance status (PM2) resulted in a significant increment of the AUC in comparison with the model without FLI, both in normoglycemic (Fig. 2A) and subjects with prediabetes (Fig. 2B). NRI and IDI values for the potential predictive improvement associated with these incremented AUCs were not significant.

No significant differences were found in any of the studied groups comparing PM1vs PM2 AUCs in models with FLI; nor in models without FLI.

## Discussion

In this cohort of the Spanish adult population, we found that FLI levels were positively associated with the risk of incident T2DM after 7.5 years follow-up, independently of different risk factors for diabetes such as age, sex, FGL, family history of T2DM, HOMA-IR, plasma lipids, hypertension or lifestyle. We also showed that the association of FLI with the development of T2DM remained within the normoglycemic Spanish population, even though the risk of diabetes in this group was lower. Moreover, we found that models including categorized FLI correctly reclassified a substantial proportion of incident T2DM cases of the Spanish overall population independently of the presence of the HOMA-IR in the prediction models.

Increasing epidemiological evidence suggests that there is a bidirectional relationship between NAFLD and T2DM which may be linked by insulin resistance^12–14^. In relation to this, FLI is not only considered a good marker for fatty liver in clinical practice, but it has also been suggested as a rough clinical estimate of abnormal insulin sensitivity and secretion^21^.

The majority of previous studies have shown that higher FLI levels might be a predictor of the development of incident T2DM in the general population^23,24,27^ and in patients with prediabetes^14,25,26^ independently of different confounding variables. According to these, in our study, in all models the highest FLI category was significantly associated with the development of incident T2DM after 7.5 years in the general population and within subjects with prediabetes. Franch-Nadal et al. have previously published the association of FLI-diagnosed hepatic steatosis with the risk of developing T2DM in the Spanish population with prediabetes at a shorter follow-up period; nevertheless, unlike our investigation, the lack of data on HOMA values did not allow them to disentangle the respective effects of HOMA-IR and FLI on the risk of T2DM development. Contrary to our findings, data from the IT-DIAB study^25^, in which the association between FLI and T2DM conversion was studied in a sample of 389 subjects with prediabetes from three different French cities, indicated that after including the HOMA levels in their multivariate analysis, the FLI was no longer significantly associated with conversion to new onset of T2DM. This difference could be potentially explained, as some of the authors declared in the study limitations, by their limited sample size and the lack of statistical power to draw adequate mixed models.

Our results also show for the first time that high FLI levels are associated with the risk of T2DM development in normoglycemic subjects from a European population. Moreover, this association was also independent of other confounding variables such as sex, age, baseline FGL, family history of T2DM, lifestyle, hypertension, lipid profile, level of transaminases and insulin resistance risk category. To the best of our knowledge, only one other study has been performed so far assessing the association of FLI levels and incidence of T2DM in normoglycemic subjects^29^. This study was performed in a Japanese population and concurs with our current results, higher FLI levels were found to be a predictor of incident T2DM in individuals without prediabetes according to a model adjusted by age, systolic blood pressure, low-density lipoprotein cholesterol, high-density lipoprotein cholesterol, smoking and alcohol consumption status. However, unlike our investigation, the impact of insulin resistance on the relationship between FLI and T2DM was not examined in their study.

In our study, FLI was able to significantly increased AUC for the future T2DM compared to the variables included in its calculation; additionally, the incorporation of FLI into traditional T2DM risk prediction models including age, sex, FGL, family background of T2DM, with and without HOMA-IR, significantly improved the prediction AUC of T2DM development after 7.5 years of follow-up in the Spanish general population. Similar results have been reported by Yadav et al.^23^ in a Japanese population where they also observed that, opposite to the FLI, the addition of BMI and/or waist circumference to the conventional T2DM prediction model did not significantly improve the AUC values. To further explore the added value of FLI as a T2DM predictor, we calculated the specific statistical metrics IDI and NRI, which are known to be more sensitive than AUC for the determination of improvement in the predictive value^30^. IDI can provide clinical information on increased sensitivity without sacrificing specificity and NRI can provide clinical information by quantifying the improvement. According to our knowledge, only the KoGES-ARIRANG Japanese study has reported information about the incremental predictive value of FLI on the risk of T2DM development with IDI and NRI data^23^. Similar to this previous work, our current study showed for the overall Spanish population that FLI improved incident T2DM subject reclassification with both significant IDI and NRI, independently of the presence of the HOMA-IR in the prediction models. Additionally, when comparing the ROC curves of models including or not the insulin resistance risk category, non-significant differences for better predicting T2DM development were found. Altogether, these data suggest that for the general Spanish population, FLI not only act as an additional contributor to predicting incident T2DM when applied with these conventional risk factors, but it could also be considered as an effective and efficient alternative to HOMA determination in clinical practice to evaluate the risk of T2DM development avoiding the cost of the circulating insulin determination.

When comparing AUCs from the ROC curves in the groups of subjects according to the prediabetes status, the improvement resulting from the inclusion of FLI in the prediction models was only significant when these models excluded HOMA-IR category. Nevertheless, non-significant reclassification improvement estimated by NRI nor IDI statistics was found after the inclusion of FLI in any of the models. No previous data have been published so far regarding the quantification of the predictive value of FLI in the development of T2DM by either using NRI or IDI methods according to the prediabetes status.

Our study presents some limitations. Even if FLI was first proposed as a good readout of hepatic steatosis^18^, we were not able to confirm hepatic steatosis in our population as relevant imaging data. Subjects with severe diseases such as hepatitis were excluded from the cohort; however, participants were not screened for other different forms of liver disease. Although participation in the follow-up was 66%, the possible participation bias was minimal^31^; nevertheless, the limited sample size when the overall population is split according to prediabetes status might be the cause of the lack of significant differences between AUCs and NRI and IDI values. Alternatively, sample size might be also limited to detect significant associations in the analysis men and women separately when overall population is split by the prediabetes status (especially in the group of subjects with prediabetes); nevertheless, RR for the association between categories of FLI and T2DM incidence were similar between men and women, which let us to believe that there were not differences between men and women and the lack of significant associations was mainly due to a limited sample size; to avoid possible interferences related to the sex, we included it as confounding variable in all the multivariate models. Prescription drugs are a well-known cause of hepatotoxicity and although the use of some of the most known steatogenic drugs has been considered in the models adjustment, the list of these medications is long and heterogeneous and it was impossible to assess the effects of other medications. There could also be an underdiagnosis in the declared interpretation of the subjects in relation to their pharmacological treatments and this cannot be ruled out. As it is common in epidemiological studies, HOMA index was used to estimate insulin resistance instead of performing the clamp method. HOMA-IR index has been validated against the gold-standard hyperinsulinaemic euglycaemic clamp (an invasive, intensive and technically difficult procedure), and is considered a reliable index to assess insulin sensitivity in epidemiological studies which assess “in vivo” insulin sensitivity in humans^32^. Even that in our results FLI showed a significantly higher predicting T2DM AUC values than its individual components, and although previous investigations have reported that the addition of BMI and/or waist circumference to conventional T2DM prediction model did not significantly improve the AUC values^23^; it is likely that the improvement observed in the AUC after the incorporation of FLI into the MP1 and MP2 models, might be related to the variables included in the FLI calculation, then this result should be carefully considered.

The main strength of the study is that the data were obtained from a large national wide-cohort, with a considerable duration of the follow-up and substantial number of events. Most of the participants underwent an OGTT to diagnose T2DM or prediabetes, and in the follow-up, HbA1c was also used, which guarantees the capture of most of the incident T2DM.

In conclusion, data gathered from this national cohort demonstrated an independent association between the high FLI category and the risk of incident T2DM. Our results also indicate that FLI, a simple surrogate measure of hepatic steatosis, may be considered as an early indicator of T2DM not only in both the general population and subjects with prediabetes, but also in subjects with normal glucose levels. Our current investigation also suggest that FLI might provide additional information for future T2DM to prediction models including the conventional risk factors sex, age, FGL and insulin resistance risk category. In addition, this index might be useful as an alternative to HOMA in clinical practice to evaluate future T2DM risk. Further investigations are needed to confirm the predictive value of FLI in groups of individuals with and without prediabetes, as well as to generalize the value of this risk-scoring tool for predicting incident T2DM.

## Methods

### Study design, setting and population

Samples and data were based on the population-based, cohort study Di@bet.es epidemiological trial.

The initial cross-sectional study of the Di@bet.es was undertaken in 2008–2010 from a random cluster sampling of the Spanish population^33^. The Di@bet.es study sample consisted of 5072 subjects more than 18 years old, randomly selected from National Health System registries distributed into 100 clusters. Subjects with severe disease such as cancer or hepatitis were excluded by protocol. The Di@bet.es cohort was re-evaluated in 2016–2017 (the follow-up time was 7.5 ± 0.6 years) and finally 2408 subjects completed the follow-up. Detailed information on the methodology of the Di@bet.es cohort study has been previously described^31^.

For the present sub-study only followed up participants at risk of T2DM (who had not T2DM at baseline) and from which FLI levels were possible to calculate were included in the analyses (n = 2260). The study population was classified according to the presence (n = 641) or absence (n = 1619) of prediabetes at baseline, which was defined as having FGL ≥ 100 mg/dl and/or post OGTT glucose level ≥ 140 and < 200 mg/dl.

The research was carried out in accordance with the Declaration of Helsinki (WHO 2011) of the Word Medical Association. Written informed consent was obtained from all the participants. The study was approved by the Ethics and Clinical Investigation Committee of the Hospital Regional Universitario de Málaga (Malaga, Spain) in addition to other regional ethics and clinical investigation committees all over Spain.

### Data collection and laboratory measurements

In both phases of the study, the participants were invited to attend an examination visit at their health centre with a nurse specially trained for this project. Information was collected using an interviewer administered structured questionnaire, followed by a physical examination and blood sampling.

For the present study the anthropometric and sociodemographic variables considered were: age, sex, weight, waist, family history of T2DM; educational level (classified as unlettered, attendance to primary or high school, and university); alcohol consumption (never: no alcohol consumption, low: < 1 serving/week, moderate: between 1 serving/week and 2 servings/day for men and 1 serving/day for women, and high: > 2 servings/day for men and over 1 serving/day for women); adherence to the Mediterranean diet (a 14-point Mediterranean diet score was calculated^31^ and the cut-off for considering adherence was over 8 points in the score); physical activity (classified as low, moderate and high levels according to the IPAQ questionnaire^34^); smoking habits (current smokers vs former/never been smokers). The use of steatogenic medications^35^ (amiodarone, methotrexate, tamoxifen, fluoxetine, valproic acid, acetylsalicylic acid or nonsteroidal anti-inflammatory drugs) has been also considered.

Also, clinical variables such as blood pressure levels, fasting levels of glucose, insulin and lipid profile (total cholesterol, high-density lipoprotein, low-density lipoprotein and triglycerides (TAG)) and transaminases (GGT, aspartate transaminase (AST) and alanine transaminase (ALT) were considered.

BMI was calculated. Insulin resistance was estimated by the homeostasis model assessment (HOMA)^36^, and the HOMA 75th percentile of our population excluding subjects with T2DM was calculated as the insulin resistance risk category (HOMA-IR).

### FLI calculation

The FLI levels were calculated as a surrogate marker of hepatic steatosis based on the measures of TAG, GGT, BMI and waist circumference, using the formula described^18^. FLI values (ranging from 0 to 100) were also classified into three categories: low FLI (levels < 30), intermediate FLI (levels between 30 and 60) and high FLI (levels > 60), as it was established to rule out (low FLI category) and confirm (high FLI category) the presence of NAFLD^18^.

### Definition of new cases of T2DM

New cases of T2DM at follow up were diagnosed as fasting serum glucose equal or higher than 126 mg/dl or 2 h post OGTT equal or higher than 200 mg/dl or HbA1c equal or higher than 6.5% or use of glucose-lowering medication at the follow-up examination^37^.

### Statistical analysis

Data are presented as means ± SD, or proportions. Differences in baseline variables according to FLI categories were measured by ANOVA adjusted by sex, age and BMI or Chi-square test. Variables not following a normal distribution were log-transformed to perform the ANOVA analyses.

Incidence rate ratios of T2DM according FLI categories were calculated in the overall population and in the group of subjects with and without prediabetes using Poisson Robust Regression models^38^ adjusted by different potential confounders (age, sex, FGL, family history of T2DM, HOMA-IR, physical activity, adherence to the Mediterranean diet, AST and ALT, steatogenic medications, alcohol consumption, educational level, hypertension and/or serum lipids levels).

The discrimination power of the proposed models with and without FLI was tested by ROC curves analysis. Differences in the ROC curves were tested by the Delong’s test. Furthermore, we used net reclassification improvement (NRI) and integrated discrimination index (IDI)^30^ to quantify the improvement in reclassification and sensitivity based on the addition of FLI to proposed models including the conventional risk factors for prediction of incident T2DM age, sex, family history of T2DM, FGL, with and without HOMA-IR.

Analyses were made using SPSS v20 (IBM, Chicago, IL, USA).

### ﻿Supplementary Information

Supplementary Information.

## Appendix

Di@bet.es-incidence Study Group:

G. Rojo-Martínez, F. Soriguer, S. Valdés, N. Colomo, C. Maldonado, E. García-Escobar, A. Lago-Sampedro and S. García-Serrano (Centro de Investigación Biomédica en Red de Diabetes y Enfermedades Metabólicas Asociadas [CIBERDEM]; UGC Endocrinología y Nutrición, Hospital Regional Universitario, Instituto de Investigación Biomédica de Málaga [IBIMA], Malaga, Spain), A. Goday (Servicio de Endocrinología y Nutrición, Hospital del Mar, Barcelona, Spain), E. Bordiú (Laboratorio de Bioquímica, Hospital Universitario San Carlos, Madrid, Spain), A. Calle-Pascual (Servicio de Endocrinología y Nutrición, Hospital Universitario San Carlos, Madrid, Spain and CIBERDEM), L. Castaño and I. Urrutia (CIBERDEM; Unidad de Investigación, Hospital Universitario Cruces, Universidad del País Vasco/Euskal Herriko Unibertsitatea [UPV/EHU], Baracaldo, Vizcaya, Spain), C. Castell (Servicio de Prevención enfermedades crónicas no transmisibles, Departamento de Salud. Barcelona, Spain, E. Delgado and E. Menéndez (Servicio de Endocrinología y Nutrición, Hospital Central de Asturias, Oviedo, Asturias, Spain), J. Franch-Nadal (Equipo de Atención Primaria Raval Sud, Institut Català de la Salut, Red GEDAPS [Grupo de Estudio de la Diabetes en Atención Primaria de la Salud], Unitat de Suport a la Recerca, Institut d’Investigació en Atenció Primària Jordi Gol, Barcelona, Spain), S. Gaztambide (CIBERDEM; Servicio de Endocrinología y Nutrición, Hospital Universitario de Cruces, UPV/EHU, Baracaldo Vizcaya, Spain), J. Girbés (Unidad de Diabetes, Hospital Arnau de Vilanova, Valencia, Spain), R. Gomis (CIBERDEM; IDIBAPS, Hospital Clínic de Barcelona, Barcelona, Spain), E. Montanya (Bellvitge Hospital-IDIBELL, University of Barcelona, Hospitalet de Llobregat, Barcelona, Spain), E. Ortega (CIBEROBN; IDIBAPS, Hospital Clínic de Barcelona, Barcelona, Spain), I. Ramis (CIBERDEM, Spain), J. Vendrell (CIBERDEM; Rovira i Virgili University; Servicio de Endocrinología y Nutrición, Hospital Universitario Joan XXIII, Institut d’Investigacions Sanitàries Pere Virgili, Tarragona, Spain).
