# Supplementary material for: Fatty liver index as a predictor for type 2 diabetes in subjects with normoglycemia in a nationwide cohort study
Source: Sci Rep. 2021 Aug 12;11:16453. doi: 10.1038/s41598-021-95546-x (PMC8361016; doi:10.1038/s41598-021-95546-x)

Fatty liver index as a predictor for type 2 diabetes in subjects with normoglycemia in a nationwide cohort study

E. García-Escobar PhD.[1,2,*], S. Valdés PhD.[1,2], F. Soriguer PhD.[1,2], J. Vendrell PhD.[1,3], I.M. Urrutia-Etxebarria[1,4,5], C. Maldonado-Araque PhD.[1,2], E. Ortega PhD.[6,7], P. Ocón PhD[8], E. Montanya PhD.[1,9], E. Menéndez PhD.[5,10], A. Lago-Sampedro[1,2], T. González- Frutos[4], R. Gomis PhD[1,6], A. Goday PhD.[7,11] , S. García-Serrano PhD.[1,2], J.L. Galán-García PhD.[12], C. Castell PhD.[13], E. Bordiú PhD.[14], R. Badía[2], G. Aguilera-Venegas PhD.[12], J. Girbés PhD.[15,+], S. Gaztambide PhD.[1,4,5,+], E. Delgado PhD.[5,10,+], F. J Chaves PhD.[1,16,+], L. Castaño PhD.[1,4,5,+], A. Calle-Pascual PhD.[1,14,+], G. Rojo-Martínez PhD.[1,2,*,+], J. Franch-Nadal PhD.[1,17+].

Supplementary Table S1. New cases of T2DM over the follow-up study according to sex and FLI categories . p^a=significant differences according to FLI categories measured by Chi-Square test. p^b=Significance level in the P for trend test.

	Men					
	New-onset of T2DM (n)	Low FLI	Intermediate FLI	High FLI	p^a	p^b
Overall population	67	1.91%	4.71%	12.22%	<0.001	<0.001
Normoglycemic subjects	19	1.13%	3.50%	4.56%	0.14	<0.01
Prediabetic subjects	48	6.25%	7.89%	21.05%	0.01	<0.001
	Women					
	New-onset of T2DM (n)	Low FLI	Intermediate FLI	High FLI	p^a	p^b
Overall population	76	1.73%	5.08%	14.90%	<0.001	<0.001
Normoglycemic subjects	18	0.62%	1.93%	5.84%	<0.001	<0.001
Prediabetic subjects	58	8.65%	12.5%	25.16%	0.001	<0.001

Supplementary Table S2. Incidence rate ratios and 95% confidence intervals of Poisson robust multivariate regression models for the risk of incident T2DM after 7.5 years of follow-up according to FLI values in the general population and according to prediabetic status. p=Significance level in the regression model.

	Overall population		Subjects with normoglycemia		Subjects with prediabetes	
	FLI	p	FLI	p	FLI	p
Base model: age, sex, fasting glucose and family history of T2DM	1.017(1.011-1.024)	<0.001	1.022(1.009-1.035)	0.001	1.013(1.006-1.021)	<0.001
Base model + HOMA-IR	1.016(1.009-1.024)	<0.001	1.021(1.007-1.036)	0.004	1.013(1.005-1.020)	0.002
Base model + Total and HDL and LDL cholesterol + Dyslipidemia treatment	1.015 (1.008-1.022)	0.001	1.021(1.009-1.033)	0.001	1.010(1.002-1.018)	0.013
Base model + AST + ALT + Steatogenic medications	1.017(1.010-1.024)	<0.001	1.020(1.006-1.033)	0.004	1.013(1.006-1.021)	0.001
Base model + Hypertension	1.018(1.010-1.025)	<0.001	1.023(1.010-1.036)	0.001	1.013(1.005-1.021)	0.001
Base model + Alcohol consumption + Educational level + Smoking habits + Mediterranean diet adherence + Physical activity	1.017(1.010-1.024)	<0.001	1.023(1.010-1.036)	0.001	1.013(1.005-1.020)	0.001

Supplementary Table S3. Analyses by sex of the incidence rate ratios and 95% confidence intervals of Poisson robust multivariate regression models for the risk of incident T2DM after 7.5 years of follow-up according to FLI categories in the general population. p^a=Significance level in the regression model. p^b= Significance level in the p for trend test. RC = reference category.

	Men (894)				
	Low FLI	Intermediate FLI	High FLI	p^a	p^b
Base model: age, sex, fasting glucose and family history of T2DM	RC	1.73(0.55-5.39)	3.39(1.22-9.40)	0.02	0.01
Base model + HOMA-IR	RC	1.70(0.54-5.37)	3.27(1.12-9.54)	0.03	0.01
Base model + Total and HDL and LDL cholesterol + Dyslipidemia treatment	RC	1.65(0.53-5.17)	2.93(1.05-8.19)	0.04	<0.05
Base model + AST + ALT + Steatogenic medications	RC	1.58(0.50-5.02)	2.87(1.02-8.12)	0.04	<0.05
Base model + Hypertension	RC	1.53(0.48-4.87)	3.22(1.14-9.07)	0.02	0.01
Base model + Alcohol consumption + Educational level + Smoking habits + Mediterranean diet adherence + Physical activity	RC	1.68(0.52-5.37)	3.48(1.24-9.74)	0.02	<0.01
	Women (1366)				
	Low FLI	Intermediate FLI	High FLI	p^a	p^b
Base model: age, sex, fasting glucose and family history of T2DM	RC	1.31(0.62-2.78)	2.77(1.41-5.45)	<0.01	<0.01
Base model + HOMA-IR	RC	1.23(0.57-2.63)	2.47(1.22-5.00)	0.01	<0.01
Base model + Total and HDL and LDL cholesterol + Dyslipidemia treatment	RC	1.23(0.58-2.61)	2.49(1.26-4.91)	0.01	<0.01
Base model + AST + ALT + Steatogenic medications	RC	1.31(0.62-2.79)	2.86(1.46-5.62)	<0.01	<0.01
Base model + Hypertension	RC	1.33(0.63-2.82)	2.92(1.43-5.97)	<0.01	<0.01
Base model + Alcohol consumption + Educational level + Smoking habits + Mediterranean diet adherence + Physical activity	RC	1.25(0.59-2.64)	2.75 (1.38-5.47)	<0.01	<0.01

Supplementary Table S4. Analyses by sex of the incidence rate ratios and 95% confidence intervals of Poisson robust multivariate regression models for the risk of incident T2DM after 7.5 years of follow-up according to FLI categories and the prediabetes status. p=Significance level in the regression model. RC = reference category.

**Subjects with normoglycemia**				
	Men (596)			
	Low FLI	Intermediate FLI	High FLI	p
Base model: age, sex, fasting glucose and family history of T2DM	RC	2.44(0.50-11.78)	3.06(0.71-13.18)	0.13
Base model + HOMA-IR	RC	2.43(0.51-11.70)	3.01(0.62-14.55)	0.17
Base model + Total and HDL and LDL cholesterol + Dyslipidemia treatment	RC	2.99(0.58-15.48)	3.39(075-15.34)	0.11
Base model + AST + ALT + Steatogenic medications	RC	2.34(0.47-11.57)	2.53(0.56-11.45)	0.23
Base model + Hypertension	RC	2.97(0.65-13.50)	2.11(0.42-10.71)	0.16
Base model + Alcohol consumption + Educational level + Smoking habits + Mediterranean diet adherence + Physical activity	RC	2.10(0.48-9.14)	2.71(0.69-10.55)	0.15
	Women (1023)			
	Low FLI	Intermediate FLI	High FLI	p
Base model: age, sex, fasting glucose and family history of T2DM	RC	1.70(0.35-8.19)	4.12(1.09-15.51)	0.03
Base model + HOMA-IR	RC	1.60(0.34-7.50)	3.46(0.98-13.56)	0.07
Base model + Total and HDL and LDL cholesterol + Dyslipidemia treatment	RC	1.74(0.37-8.08)	4.50(1.39-14.60)	0.01
Base model + AST + ALT + Steatogenic medications	RC	1.63(0.34-7.83)	3.84(0.97-15.06)	0.05
Base model + Hypertension	RC	1.70(0.35-8.15)	4.16(1.09-15.86)	0.04
Base model + Alcohol consumption + Educational level + Smoking habits + Mediterranean diet adherence + Physical activity	RC	1.70(0.38-7.64)	4.54(1.23-16.83)	0.02
**Subjects with prediabetes**				
	Men (298)			
	Low FLI	Intermediate FLI	High FLI	p

Base model: age, sex, fasting glucose and family history of T2DM	RC	1.15(0.24-5.42)	2.78(0.69-11.10)	0.15
Base model + HOMA-IR	RC	1.14(0.24-5.53)	2.73(0.65-11.52)	0.17
Base model + Total and HDL and LDL cholesterol + Dyslipidemia treatment	RC	1.06(0.23-4.84)	2.22(0.61-8.99)	0.22
Base model + AST + ALT + Steatogenic medications	RC	1.06(0.23-4.93)	2.31(0.57-9.39)	0.24
Base model + Hypertension	RC	1.07(0.23-4.94)	2.58(0.66-10.16)	0.17
Base model + Alcohol consumption + Educational level + Smoking habits + Mediterranean diet adherence + Physical activity	RC	1.11(0.22-5.53)	2.67(0.64-11.22)	0.18
	Women (343)			
	Low FLI	Intermediate FLI	High FLI	p
Base model: age, sex, fasting glucose and family history of T2DM	RC	1.02(0.45-2.32)	2.05(1.05-4.01)	0.03
Base model + HOMA-IR	RC	0.99(0.42-2.26)	1.89(0.92-3.89)	0.08
Base model + Total and HDL and LDL cholesterol + Dyslipidemia treatment	RC	0.92(0.40-2.08)	1.71(0.84-3.48)	0.14
Base model + AST + ALT + Steatogenic medications	RC	1.02(0.45-2.32)	2.26(1.17-4.38)	0.16
Base model + Hypertension	RC	1.06(0.46-2.43)	2.28(1.07-4.86)	0.03
Base model + Alcohol consumption + Educational level + Smoking habits + Mediterranean diet adherence + Physical activity	RC	1.03(0.46-2.30)	1.94(0.98-3.83)	0.06

Supplementary Figure S1. Comparison of area under the receiver-operating characteristic curve (AUC) for incident T2DM in overall population between FLI and its individual components: BMI (A), waist circumference (B), serum GGT levels (C) and serum triglycerides levels (D).

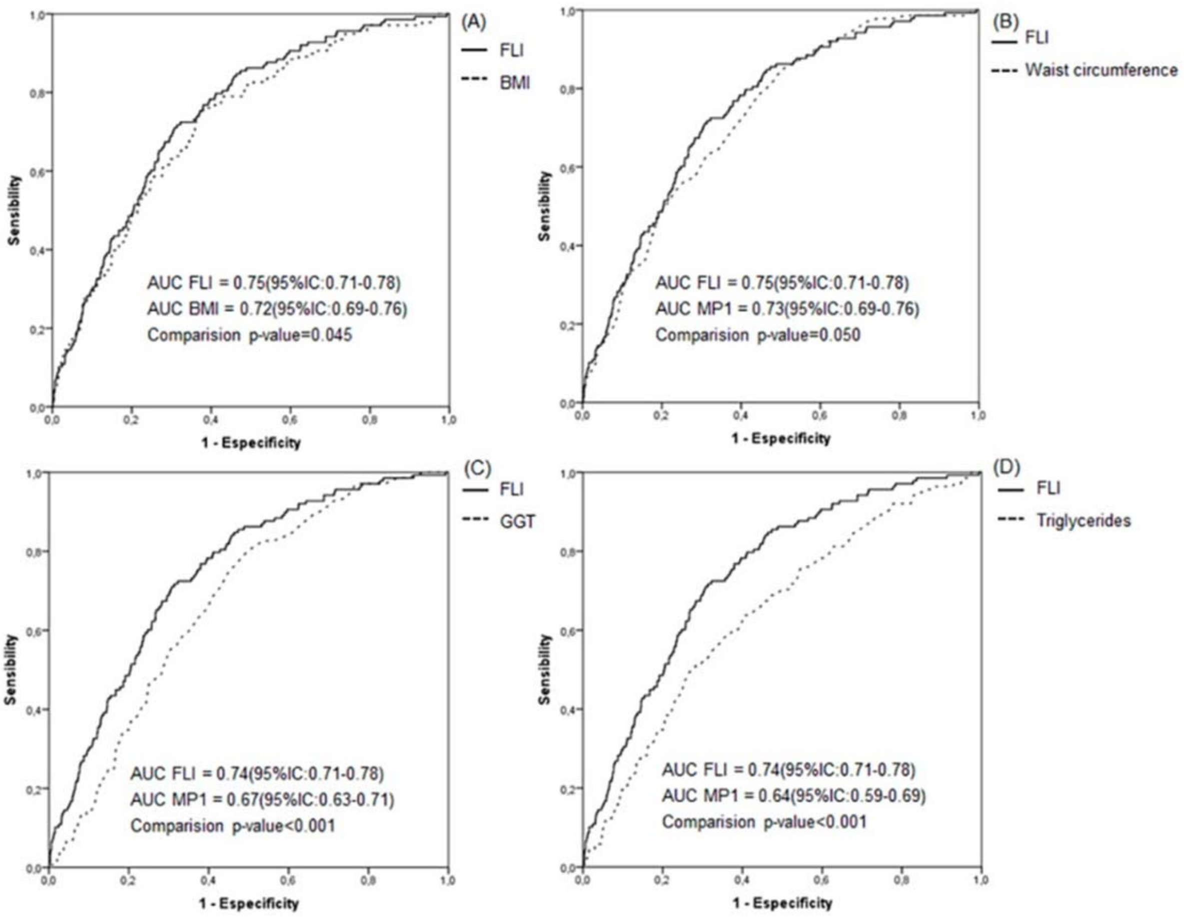

Supplement: Supplementary file 1 — Supplementary Information. [file 41598_2021_95546_MOESM1_ESM.pdf]
